# New Data for Nebivolol after In Silico PK Study: Focus on Young Patients and Dosage Regimen

**DOI:** 10.3390/pharmaceutics14091911

**Published:** 2022-09-09

**Authors:** Lara Marques, Bárbara Costa, Nuno Vale

**Affiliations:** 1 OncoPharma Research Group, Center for Health Technology and Services Research (CINTESIS), Rua Dr. Plácido da Costa, 4200-450 Porto, Portugal; 2 CINTESIS@RISE, Faculty of Medicine, University of Porto, Al. Prof. Hernâni Monteiro, 4200-319 Porto, Portugal; 3 Department of Community Medicine, Health Information and Decision (MEDCIDS), Faculty of Medicine, University of Porto, Rua Dr. Plácido da Costa, 4200-450 Porto, Portugal

**Keywords:** Nebivolol, in silico pharmacology, population PK, dosage regimen

## Abstract

Nebivolol (NEB) is a highly selective β1 receptor antagonist with a distinct pharmacological profile. This drug is approved for the treatment of hypertension in the US, and hypertension and heart failure in Europe. Here, we review observations based on age dependence and explore new drug regimens with in-silico studies, to achieve better efficacy and safety. The clinical data were obtained from six published literature reports. Then the data were used for model building, evaluation, and simulation. A two-compartment model with first-order absorption, lag time, linear elimination, and the following covariates: age and genotype were the ones best describing our population. Simulation of different dose regimens resulted in an increase chance of efficacy and safety when the dose regimen was altered to 6 mg every 36 h. It is worth noting that our population in this study constituted of young and healthy individuals. Studies regarding the effects of NEB according to age are scarce; however, they are needed to further improve efficacy and safety, and reduce adverse effects.

## 1. Introduction

**Figure 1:**
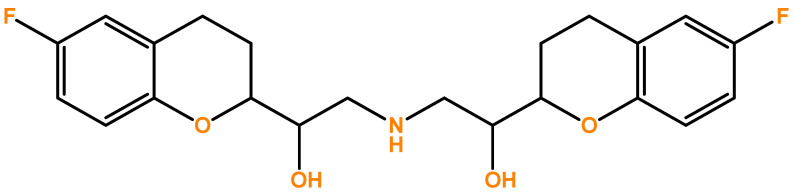
Chemical structure of drug Nebivolol (NEB).

Nebivolol (NEB, Figure 1) is a third-generation β-blocker drug, a β-adrenergic receptor antagonist with high selectivity for β1-adrenergic receptors. In addition, it has a vasodilating effect mediated by the endothelial L-arginine/nitric oxide (NO) pathway [1,2]. This dual mechanism, responsible for the haemodynamic profile, explains the prominence given to this drug within the β-blocker family [3,4]. It plays a key role in cardiovascular therapy, having been widely used for hypertension and chronic heart failure treatments. In fact, its ability to prevent increased pulse rate and control heart pumping is reported, in addition to its vasodilating action on blood vessels [2].

NEB was developed in the 1980s and its clinical use in Europe began in 1997 [5]. A few years later, in 2007, Food and Drug Administration (FDA) approved the drug [6]. NEB should first be taken once a day at a starting dose of 5 mg, and depending on each patient’s blood pressure, the dosage must be adjusted. The dose can be gradually increased at intervals of 2 weeks, up to a maximum dose of 40 mg per day. The starting dose can be decreased to 2.5 mg once daily in patients with mild renal insufficiency or hepatic impairment. 

Pharmacokinetic parameters documented in the literature demonstrate that NEB is rapidly absorbed (1.5–4 h) after a standard 5 mg dose intake. Its metabolism occurs in cytochrome P450 (CYP) 2D6, which is a drug-metabolizing enzyme that is regularized by genetic polymorphism. Therefore, the bioavailability of NEB ranges from 12% in extensive metabolizers (EMs, people with normal rate of metabolism) to 96% in poor metabolizers (PMs, people with slow rate of metabolism). Moreover, it is known that the metabolism pathways can differ according to the phenotype, yet both phenotypes demonstrate the same clinical effects [1,2]. As well as genotype, age is not considered to be an interference factor of NEB pharmacokinetics [2], however, studies regarding age and sex differences in the efficacy and safety of NEB are scarce. 

Among all β-blockers, NEB exhibits the greater tolerability, with few recorded adverse effects. The most common ones are headache, fatigue, paraesthesia, and dizziness. Bradycardia, Atrioventricular block, and Raynaud’s syndrome may occur when the medical prescription includes a larger dose. Furthermore, when the drug is able to penetrate the blood-brain barrier, adverse effects on the central nervous system (CNS) may be felt, despite being rare [5]. Even though NEB is generally safe and well tolerated, there is a great deal of interest in minimizing adverse effects so that the patients’ quality of life is improved. 

In recent decades, in silico modelling is increasingly trending, due to its numerous contributions to extending the knowledge of pharmacodynamics and pharmacokinetics [7]. The development of a population pharmacokinetic model (PK) is useful not only at an a priori stage (for example, before clinical trials begin) but also for drug optimization (even after drug approval). The purpose of this study is to develop a PK model of NEB based on the literature data and optimize the dose regimen administered in patients diagnosed with cardiovascular disease. The optimization involves finding a lower dose, ensuring equal or greater efficacy and safety than existing therapeutic regimens. The NEB PK model developed and proposed by us will follow the same assumptions as models already published in PK studies [6].

## 2. Materials and Methods

### 2.1. Literature Data Collection

PubMed database was searched for clinical PK data of NEB from its pharmaceutical approval until 30 April 2022. The keywords used for this research were «Pharmacokinetic parameters and NEB». Publications that met the established inclusion and exclusion criteria were eligible for our study. Inclusion criteria were: (1) the specific drug was NEB or NEB hydrochloride, (2) the research subjects were humans, who participated in clinical trials, (3) clinical trials must contain detailed information about study design and demographics, (4) the literature reported the plasma NEB concentration-time profiles. Exclusion criteria were: (1) study did not clearly describe the information required. 

The data (study design, inclusion and exclusion criteria, pharmacokinetic parameters, and plasma concentration-time profiles) were collected, including the concentration and time values, extracted from plots using WebPlot Digitizer (Version 4.5, Ankit Rohatgi, Pacifica, California, USA) [8]. 

### 2.2. Population Pharmacokinetic Modelling

Population PK analysis was performed using Monolix Suite 2021R1 (Lixoft, Antony, France). The estimation of population PK parameters was conducted by maximum likelihood using Stochastic Approximation Expectation-Maximization (SAEM) algorithm. 

The final population PK model is the result of a combination of structural and statistical models. The structural PK models are constituted of one-, two- or transit-compartments systems with linear elimination, whereas the statistical PK models comprise systems where individual PK parameters were assumed to follow normal distributions, with no random effects applied. The covariates tested included age, genotype, and body mass index (BMI). 

### 2.3. Model Evaluation and Validation

Bayesian Information Criterion (BIC), the precision of estimates, and the goodness-of-fit (GOF) allowed the final decision for selecting the best population PK model. The model with the lowest BIC was used to select the best model between the different alternatives (Supplementary material). Plus, complementary criterion such as Akaike information criterion (AIC) and Objective function value (OFV) were used. AIC was used to estimate the quality of each model to the others, lower AIC models indicated a better fit model. OFV was used to find the smaller values which were representative of the best fit. In our case, the values were negative, in which larger negative numbers represented a better fit than smaller negative numbers. Using these 3 parameters (BIC, AIC and OFV) we selected the model based on the minimization of these parameters. The estimated population parameters’ standard errors and the random effects error models’ standard errors were computed. During the model construction process, several diagnostic plots, including observed versus population-predicted NEB concentrations and observed versus individual-predicted NEB concentrations, were used to visually test the model’s fit. Additionally, plots of residuals and conditionally weighted residuals against time or population-predicted NEB concentrations were visually inspected.

The covariates tested included age, genotype, and BMI. To compare random effects to covariates, we used statistical tests included into Monolix that used specific values taken from the conditional distribution. Using the ANOVA for categorical covariate and the Pearson’s correlation test for continuous covariate, a *p*-value can be calculated. Regardless of whether they are incorporated into the model or not, the random effect-covariate associations are sorted using the *p*-values. To make decisions, both forward and backward selection was used. Until there are no correlation *p*-values over a threshold, the covariate with the smallest correlation *p*-value is included to the model, or the next smallest if the smallest has already been attempted. Until there are no correlation *p*-values below a threshold, the covariate with the highest (least significant) correlation *p*-value is eliminated, or the next highest if the highest has already been attempted. Therefore, ovariates that improved the fit while lowering BIC remained in the model. The covariates with a *p*-value less than 0.05 were considered. The predictive power of the generated covariate model was then evaluated numerically and graphically.

### 2.4. Monte Carlo Simulation and Optimization of NEB Dose Regimen

The PK profile of NEB in different dosing regimens was simulated using the individual PK parameter values from the literature data. First, we conducted, in a population of 1000 patients, the standard treatments for hypertensive patients and the recommended dose for people with comorbidities [1]. Next, four new dosing regimens of NEB (Table 1) were proposed, with an established threshold interval of efficacy and safety (between 1.0 and 2.0 ng/mL and below 0.5 ng/mL, respectively). Efficacy and safety targets were established through an extrapolation of information collected from literature. All simulations were performed using Simulx. In all simulations, the total duration of treatment was 15 days, as it is reported therapeutic effects after 1–2 weeks.

## 3. Results

### 3.1. Collection of the Literature Data

The clinical data, including the concentration-time profiles, were obtained from 6 published literature reports (Table 2). From this compendium, 6 virtual patients were created and real data from 24 individuals were used. Virtual patients were created based on the mean values of the demographic and PK parameters, in order to overcome the lack of information about NEB. All studies were conducted in healthy adults (and non-smoking). The dose of NEB ranged from 5 mg to 20 mg and the periods of treatment consisted of a single oral dose administration or an oral daily dose for 27 days.

### 3.2. Subject Characteristics

The demographic characteristics are summarized in Table 3. A total of 30 patients, 5 receiving a single oral dose of 5 mg, 24 receiving a single oral dose of 10 mg, and 1 receiving an oral daily dose of 20 mg, were included in this study. The mean age and BMI are 26.70 and 25, respectively. Furthermore, our sample is divided into three groups, according to genotype: EMs represent 56.7% of total patients, followed by intermediate metabolizers (IMs), with 40%; there is only one with poor metabolization. The ethnicities represented in this group of patients are Caucasian and Asian. 

**Figure 2:**
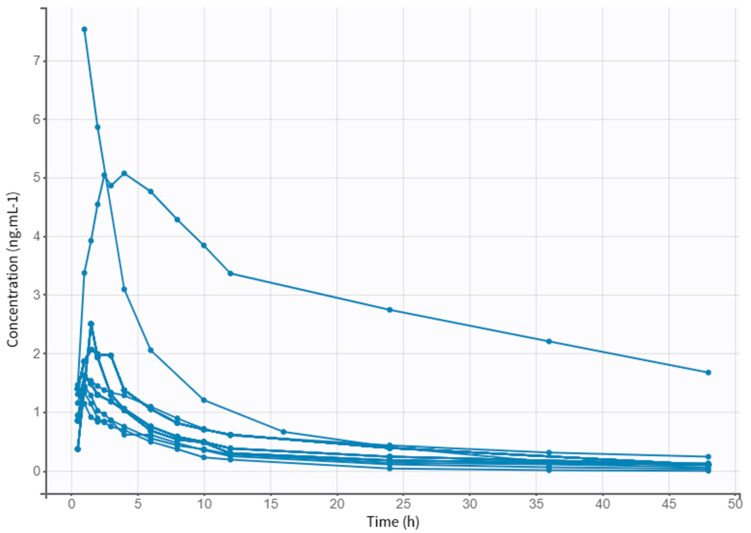
Observed data of plasma NEB concentration (ng·mL^–1^) through time (h).

A preliminary analysis of clinical data allowed the collection of PK parameters of different doses of NEB. Table 4 displays the plasmatic peak (C*max*), the area under the curve (AUC), the time at which the C*max* is reached (T*max*), the rate at which drug is removed from the human body (K*el*), and the half-life (t_1/2_). The obtained values follow the same values described in the literature for the different doses. Figure 2 shows the evolution of drug concentration over time in each subject for a period of 48 h. 

### 3.3. Model Building Process

The basic model that best described the population pharmacokinetic profile, as determined by the smallest BIC value, is a two-compartment model with first-order absorption, lag time, and linear elimination. A proportional error model and normal parameter distribution were used to develop this model. The covariate analysis demonstrated an effect of age on clearance (Cl) and genotype has a significant impact on Cl, volume of distribution (V_d_), and lag time (T*lag*). Adding other covariates did not further significantly improve the model. Table 5 displays the parameters estimates of the final model.

### 3.4. Model Evaluation

**Figure 3:**
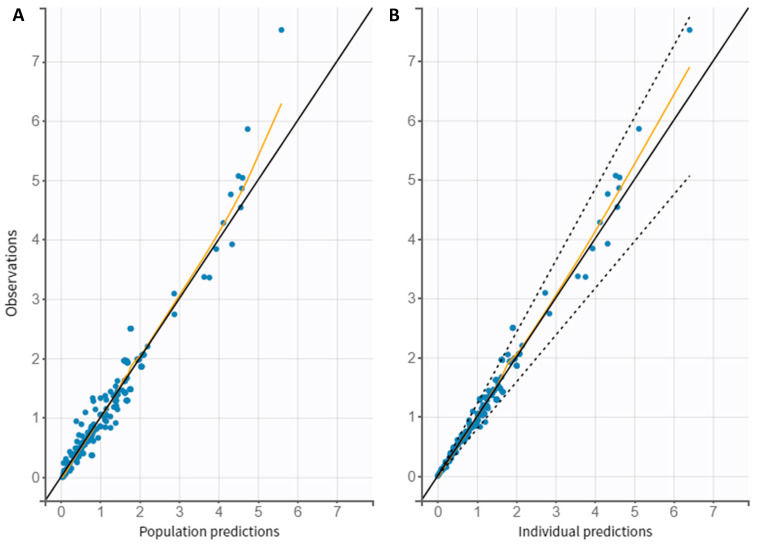
Final NEB covariate pharmacokinetic model. (**A**) Observation vs. Population predictions, and (**B**) Observations vs. Individual predictions of NEB concentration (ng/mL). The line of identity is visualized as a solid black line. The yellow line represents the spline of the data, and the 90% prediction interval is shown as a dotted line.

**Figure 4:**
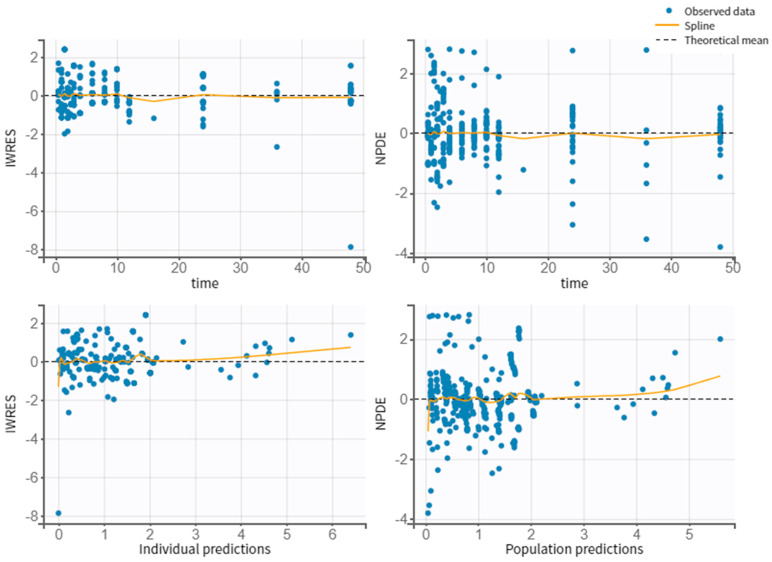
Individual weighted residuals (IWRES) and normalized predictive distribution errors (NPDE) versus time and the individual predicted concentrations.

**Figure 5:**
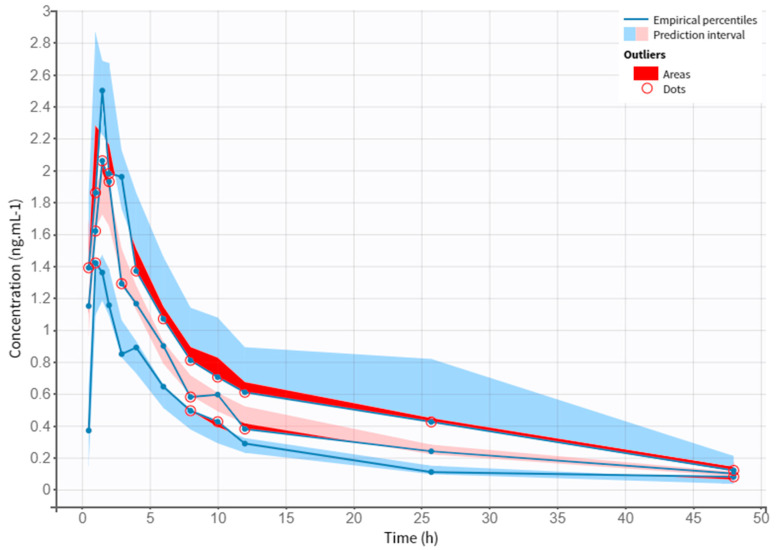
Visual Predictive Check (VPC) plot versus time using the final covariate pharmacokinetic model. The figure displays the VPC with the prediction intervals for the 10th, 50th, and 90th percentiles (from bottom to top, respectively). Outliers are visualized as red dots and areas.

Figure 3 shows the correlation between observed vs. individual or population predicted concentrations, where it can be verified that there are no outliers. Thus, the model does not present misspecifications. Figure 4 represents the scattered plots of residuals individual weighted residuals and normalized prediction distribution error metrics revealed a random distribution centered on zero. The Visual Predictive Check (VPC) plot demonstrates the fit of the simulated observations within the 90% prediction interval, which indicates a good agreement between the observed and simulated NEB concentration values (Figure 5).

### 3.5. Dose Regimen Optimization

**Figure 6:**
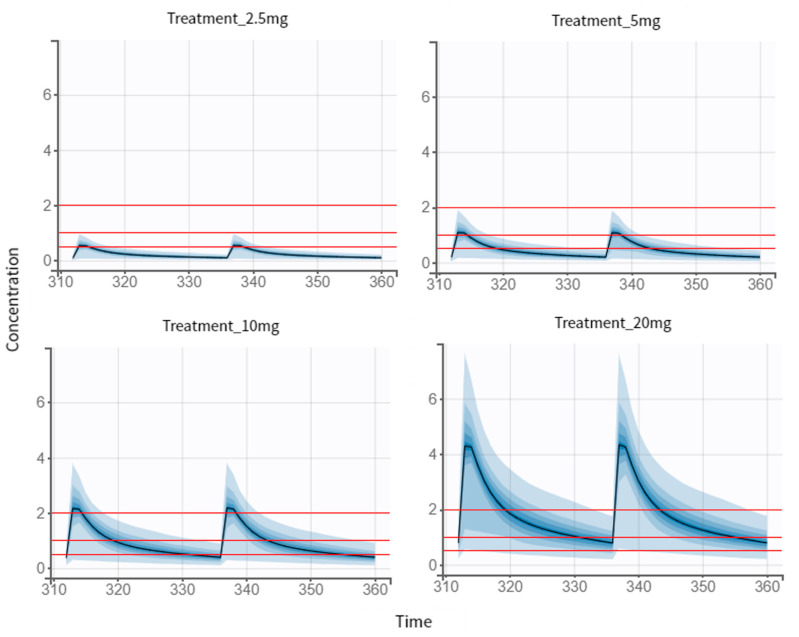
Simulations of the standard treatments of NEB. Output distributions that represent the variability between individuals with visual cues of the concentration prediction intervals. The visual cues demonstrating the efficacy (1–2 ng/mL) and safety (0.5 ng/mL) are displayed as red lines.

With the PK model developed, the optimization of the NEB therapeutic regimen had as its last step simulations of different scenarios. Peak concentrations within 1.0–2.0 ng/mL were defined as threshold of the efficacy target, and concentration below 0.5 ng/mL was defined as threshold of the safety target. First, we simulated the standard treatments for hypertensive patients (Figure 6), with the establishment of three different groups. For the 5 mg dose regimen (reference dose), we observed a 72.2% of chance of being efficient and a 95.5% of chance of being safe were observed for an individual of this population. Regarding the 10 mg dose regimen, 22.6% and 69.6% were the identified probabilities for efficacy and safety targets, respectively. The highest simulated dose (20 mg), according to our results, was the one with the lowest efficacy and safety problems (0.5% and 19.1%, respectively), as we can observe in Table 6. 

The 2.5 mg dose regimen is the recommended initial dose for elderly patients as well as patients with renal impairment (Figure 6). According to our results, there is a 1% of chance of being efficient and 97% of being safe for an individual of this specific population, composed of young and healthy individuals, for the selected dose of regimen. In this case scenario, when comparing all existing treatments, the one that best fits the efficacy and safety criteria was the 5 mg dose regimen. Then this dose regimen was used as a reference for further studies.

#### 3.5.1. Multiple-Dose Treatment of 2.5 mg

**Figure 7:**
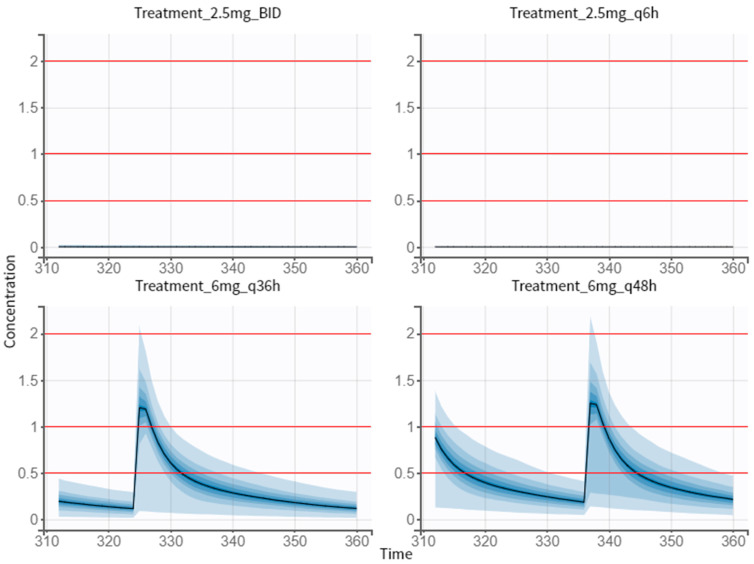
Simulations of dose regimen of 2.5 mg twice a day (Treatment 2.5 mg BID), 2.5 mg every 6 h (Treatment 2.5 mg q6 h), 6 mg every 36 h (Treatment 6 mg q36 h), and 6 mg every 48 h (Treatment 6 mg q48 h). Output distributions that represent the variability between individuals with visual cues of the concentration prediction intervals. The visual cues demonstrating the efficacy (1–2 ng/mL) and safety (0.5 ng/mL) are displayed as red lines.

As our first proposals, we tried to simulate 2.5 mg of dose in different therapeutic regimens (twice a day, BID, and every 6 h, q6 h) to investigate which of them met the efficacy and safety criteria (Figure 7). For this, the same individual parameters in all groups were ensured and the 5 mg dose regimen was the reference group when comparing the different treatments. The obtained results allowed us to conclude that 2.5 mg of dose, either twice a day or four times a day, does not reach the efficacy target (Table 7).

#### 3.5.2. Proposed Treatment of 6 mg

Increasing the amount of drug and the dosing interval was the best approach, in terms of efficacy and safety (Figure 7). Comparing to the reference group, taking a 6 mg pill every 36 h is more efficient and safer (Table 7). Likewise, the values obtained for the efficacy and safety target for the 6 mg q48 h treatment were better. Between these two simulated dose regimens, the 6 mg q36 h treatment is the most efficient and safest. 

## 4. Discussion

The European Society of Cardiology and the European Society of Hypertension guidelines presently support β-blockers for the treatment of hypertension, especially in elderly individuals, and consider that they are effective for the treatment of hypertension. Although their efficacy is age-dependent, β-blockers, like other classes of anti-hypertensives, have been demonstrated to considerably lower the risk of stroke, heart failure, and severe cardiovascular events in both younger and older patients with hypertension. In comparison to other anti-hypertensives, NEB, a β-blocker with unique properties, has shown comparable or superior therapeutic response, as well as much improved tolerability [15].

One in eight adults between the ages of 20 and 40 have hypertension. Calcium channel antagonists, angiotensin-converting enzyme inhibitors, thiazide diuretics, angiotensin receptor blockers ARB, and -blockers are all just as effective in lowering cardiovascular events in both older people and younger people under the age of 65 [16]. However, observations evaluating the efficacy and safety based on age dependence are limited.

NEB is a third-generation, highly selective β1 adrenoreceptor antagonist with NO-mediated vasodilatory effects. NEB therapy lowers systolic and diastolic blood pressure (SBP and DBP) more than other β-blockers do because many patients receive thiazide diuretics as their initial therapy, NEB and other β-blockers are typically not used as first-line medicines. The distinct characteristic of NEB is that its pharmacokinetic features are dependent on genetic polymorphism, related to cytochrome P450IID6 [17]. In the context of our study, which included pharmacokinetic data of 30 patients derived from literature, a two-compartment model with first-order absorption, lag time, and linear elimination was developed to describe the time course of NEB plasma concentration. The obtained model is consistent with the results of other publications on PK modeling of NEB [6]. For the assessment of covariate impacts on PK parameters, the following covariates were included: age and genotype; age and genotype having an impact on NEB clearance, and genotype having an impact on volume distribution and lag time. The sex covariate was not possible to include since the articles where we collected our data did not graphically discriminate which sex each individual was. Nevertheless, our model was the most complete when adding age and genetic covariates and we can assume that the PK profile of NEB may be dependent on those factors [11,14]. Although no different clinical effects seem to be documented, and contradictory literature still exists, making it difficult to determine the extent of these dependencies. 

It is widely described that NEB undergoes extensive hepatic metabolism through CYP2D6, a metabolizing-enzyme characterized by genetic polymorphisms. Therefore, our study also included individuals that had their genotype determined. Hence, it was possible to visualize the differences of efficacy and safety between the different metabolizers in study (EM, IM, and PM). Our model was able to describe higher plasma peaks in PMs (individuals with a slower biotransformation and, consequently, a marked accumulation of specific drug substrates), compared to EMs and IMs (individuals with extensive or normal enzymatic activity) [11]. No significant differences between EMs and IMs are observed in this study, as Luo et al. reported. Interestingly, different metabolizers follow a different metabolic pathway, however, the lower concentration of unchanged drug in EMs is balanced by the formation of active metabolites (4-hydroxy-nebivolol), which explains the same therapeutic effects observed in the individuals. 

As mentioned above, our model had a better fit when including age as a covariate. In our perspective, there is a clinical relevance of including age, however, the scientific community still struggles to understand the extent of its effect on different individuals. In 2013, a study with young adult patients (<55 years) with elevated diastolic blood pressure (DBP) was performed. NEB medication led to considerable improvement in BP management. Regardless of gender, color, ethnicity, hypertension stage, obesity status, and metabolic syndrome status, a substantial endpoint effect on DBP and systolic blood pressure (SBP) was seen. NEB’s pharmacologic profile, which consists of high 1 selectivity and nitric oxide-dependent vasodilatory characteristics produced by activation of 3 receptors in the endothelium, may be appropriate for the genesis of hypertension in younger patients. Raised sympathetic tone and improperly elevated peripheral vascular resistance are linked with elevated blood pressure in younger patients, who also appear to have a little greater cardiac output. NEB’s long-term safety effects, including metabolic changes, have yet to be fully understood. In this trial, patients had high prevalence of obesity and metabolic syndrome, which is not representative of a general population (plus a younger person with hypertension is expected to be burdened by other cardiovascular risk factors as well) [18].

According to previous research, men under 65 years of age have a larger incidence of hypertension than women in the same age group. However, after the sixth decade of life, women have a higher prevalence of hypertension than men. A recent study, evaluating the efficacy and safety of NEB in Korean patients with hypertension by age and sex, has reported similar data. It was demonstrated that women and elderly patients received much less NEB overall than the other research participants. In Korean clinical practice, older patients are given lower doses of NEB than younger patients to account for possible renal impairment issues with ageing (even in the absence of disease), which could result in reduced drug clearance and ultimately have the same effect as a higher dose of the drug despite the lower dose [19]. Moreover, according to Cohen-Solal et al., a distinction between patients with moderate renal impairment and patients with normal or mild renal impairment demonstrated a comparable safety profile (except from a slightly significant increase in bradycardia) [20]. Finally, body mass index, in turn, does not show NEB pharmacokinetic interference [21]. 

In this article, we also proposed a different dose regimen. NEB is administered from 5 mg to 40 mg [1]. In our study, we simulated three standard dose regimens (5, 10, and 20 mg) and the recommended dose for renal impairment (and with other comorbidities) patients (2.5 mg). The results demonstrate that taking a 5 mg tablet every day is the best option in terms of efficacy and safety. As expected, treatments that require 20 mg tablets are less safe and effective, whereas 2.5 mg dose regimen, despite having less efficacy, is safer. Comparing all the existing treatments, the one that best fits the efficacy and safety criteria was the 5 mg dose regimen for this specific population, which as a matter of fact, is the most documented in the literature. 

An optimum dosing schedule is important to provide the best quality of life to patients. Headache, fatigue, paraesthesia, and dizziness are the most common adverse effects when prescribing NEB (whether it is 5 mg or 40 mg). Since there are still associated adverse effects, our study focused on achieving a lower dose and/or altering the intake interval, ensuring equal or greater efficacy and safety than existing treatments. The first simulations included the 2.5 mg twice a day and 2.5 mg every 6 h. However, as expected these dose regimens were not efficient for this specific population of young and healthy individuals. The simulation of 2.5 mg was useful to understand how therapy can be regulated in period of dose adjustments. As mentioned above, a young patient suffering from hypertension usually has other comorbidities associated to their state, and it is important to ensure the efficacy and safety of treatments. If in this young and healthy population we obtained no efficacy, however, we could achieve a 99.1% and 100% chance of being safe for 2.5 mg BID and 2.5 mg q36, respectively. Therefore, we can assume that if a patient improves his health status (recovering from a variety of comorbidities), prescribing 2.5 mg of NEB is not effective and this dose needs to be adjusted, since these low concentrations would not be enough. Briefly, this simulation allowed us to highlight the importance of dose adjustments in clinical practice.

In the second simulation presented here, we increased the amount of drug and the dosing interval. We simulated the oral intake of 6 mg every 36 h and 6 mg every 48 h. Compared to the reference group (5 mg once a day), the dose regimen of 6 mg q36 h revealed the best efficacy and safety. Although it has a greater amount of drug, as it is administered at a greater interval, the concentration is reduced. The percentage of being safe is closer to 100%, in comparison to the 5 mg tablet, and this increase in safety could be significative. Regarding efficacy, there was an increase of 5.4% between the two treatments. These authors are aware that there is no commercialization of NEB in a tablet of 6 mg.

Clinical management is complicated due to the variations in hypertension’s type, severity, and prevalence between age groups. For instance, younger age is a significant predictor of poor therapy adherence, although older age is also related with an increased prevalence of comorbidities and drug combination. To our knowledge, there are a few studies regarding NEB efficacy and safety either in young or older patients with hypertension. It is necessary trials reporting the effects of NEB treatment, in short or long term, based on patients’ age, while available data indicates that treatment with older-generation -blockers, such as atenolol, is associated with a reduction of cardiovascular morbidity and mortality in younger (60 years) but not older patients with uncomplicated hypertension. In addition, other studies report a change in the adverse event (AE) profile with patients’ ageing, (e.g., dizziness and fatigue appear to lose and gain prominence with age, respectively). Given that the physiological reaction to medication is anticipated to differ between younger and older people, this potential trend seems scientifically feasible. However, Germino et al. concluded in their study that, although age may have an impact on the dose dependence of some AEs, these observations could be due to the division of the pooled sample [22].

Overall, evidence shows that NEB has more benefits for people older than 65, either by controlling comorbidities or by preserving cardiac function. At younger ages, information regarding NEB use is scarce, and the age-dependence of this drug is not well documented. Although it is thought to have no dependence, literature can be contradictory in this matter. 

## 5. Conclusions

NEB is effective in lowering BP either in monotherapy or in combination and has a distinct pharmacological profile. Its main characteristic is the different effect on bioavailability on different metabolizers (EM, IM, PM). The age-dependence effect of this drug has been studied less. Data regarding this matter is scarce, and the reduction of AEs is difficult if these details are not well described. In our study, we demonstrated that a 6 mg dose q36 h has better chances of being effective and safe in comparison with other regimens. Further research is necessary to understand the full mechanism of this drug and the covariates affecting its efficacy and safety. 

## Figures and Tables

**Table 1:** NEB dosing regimens simulated.

No. Treatment	Dose Regimen for NEB
1	5 mg once a day for 15 days
2	10 mg once a day for 15 days
3	20 mg once a day for 15 days
4	2.5 mg once a day for 15 days
5 *	2.5 mg BID for 15 days
6 *	2.5 mg q6 h for 15 days
7 *	6 mg q36 h for 15 days
8 *	6 mg q48 h for 15 days

* Proposed dosing regimens. BID—twice a day; q6 h—every 6 h; q36 h—every 36 h; q48 h—every 48 h.

**Table 2:** Summary of published literature reports on NEB pharmacokinetics.

Study Number	Reference	Studied Population	NEB Doses	Period of Treatments	Number of Individuals Generated
1	[9]	Healthy adults (*n* = 30)	20 mg	Oral daily dose for 27 days	1
2	[10]	Healthy adults (*n* = 23)	5 mg	Single oral dose	1
3	[11]	Healthy adults (*n* = 43)	5 mg	Single oral dose	2
4	[12]	Healthy adults (*n* = 18)	5 mg	Single oral dose	1
5	[13]	Healthy adults (*n* = 18)	5 mg	Single oral dose	1
6	[14]	Healthy adults (*n* = 24)	10 mg	Single oral dose	24
Total					30

**Table 3:** Demographic characteristics (mean ± standard deviation) of patients from literature reports.

Parameter	NEB (*n* = 30)
Age (years)	26.70 ± 1.40 (20–34 years)
Body-mass index (kg/m^2^)	25.00 ± 1.00
Genotype (*n*, %)	
Extensive Metabolizers (EMs)	17, 56.7
Intermediate Metabolizers (IMs)	12, 40
Poor Metabolizers (PMs)	1, 3.3
Ethnic (*n*, %)	
Caucasian	6, 20
Asian	24, 80

**Table 4:** PK parameters of the different doses of NEB.

Parameter	Mean	RSE (%)
5 mg		
C*max* (ng·mL^−1^)	2.40	0.78
*AUC* (ng·h·mL^−1^)	37.29	1.09
*Tmax* (h)	1.66	18.77
K*el* (L/h)	0.17	0.16
t_1/2_ (h)	6.92	5.31
10 mg		
C*max* (ng·mL^−1^)	2.65	ND
*AUC* (ng·h·mL^−1^)	1.51	ND
*Tmax* (h)	21.56	ND
K*el* (L/h)	2.72	ND
t_1/2_ (h)	12.52	ND
20 mg		
C*max* (ng·mL^−1^)	8.02	3.47
*AUC* (ng·h·mL^−1^)	41.50	0.67
*Tmax* (h)	1.32	29.76
K*el* (L/h)	ND	ND
t_1/2_ (h)	11.03	2.12

RSE—Relative Standard Error; C*max*—maximum observed concentration; *AUC*—area under the curve from the time of dosing to the last measurable positive concentration; T*max*—time of maximum observed concentration; K*el*—elimination rate constant; t_1/2_—half-life time.

**Table 5:** Estimates of the population pharmacokinetic parameters from the final model.

Parameter	Estimate	RSE (%)
T*lag* pop	0.30	10.8
K_a_ pop	2.06	10.4
C*l* pop	0.22	36.7
V1 pop	4.21	4.96
Q pop	0.59	5.73
V2 pop	7.12	11.2
Error model parameters		
b	0.13	4.63

RSE: Relative standard error; Tlag—Lag time; Ka—Absorption constant rate; C*l*—Clearance; V1 and V2—The volume of distribution of the compartments one (central) and two (peripheral); Q—intercompartmental clearance.

**Table 6:** Values of the efficacy and safety target for standard treatments for cardiac patients and recommended treatment for patients with renal impairment.

Target	2.5 mg	5 mg	10 mg	20 mg
Efficacy Target	1%	72.2%	22.6%	0.5%
Safety Target	97%	95.5%	69.6%	19.1%

**Table 7:** Values of the efficacy and safety target for each group of simulation.

Target	2.5 mg BID	2.5 mg q6 h	6 mg q36 h	6 mg q48 h
Efficacy Target	0.9%	0%	75.2%	72.6%
Safety Target	99.1%	100%	97%	96.7%

## Data Availability

Not applicable.

## Supplementary Materials

The following supporting information can be downloaded at: https://www.mdpi.com/article/10.3390/pharmaceutics14091911/s1, Table S1: Demographic characteristics (mean ± standard deviation) of patients in each clinical study; Table S2: Pharmacokinetics (PK) parameters (mean ± standard deviation) of patients in each clinical study; Table S3: Different basic population pharmacokinetic models of Nebivolol.

## Abbreviations

Nebivolol (NEB), Nitric oxide (NO), Food and Drug Administration (FDA), Cytochrome P450 (CYP), Extensive metabolizers (EMs), Poor metabolizers (PMs), Intermediate metabolizers (IMs), Central nervous system (CNS), Pharmacokinetic model (PK), Stochastic Approximation Expectation-Maximization (SAEM), Bayesian Information Criterion (BIC), Goodness-of-fit (GOF), Visual Predictive Check (VPC), Individual weighted residuals (IWRES), Normalized weighted residuals (NPDE), Twice a day (BID), Diastolic blood pressure (DBP), Systolic blood pressure (SBP), Adverse event (AE).
