# Supplementary material for: New Data for Nebivolol after In Silico PK Study: Focus on Young Patients and Dosage Regimen
Source: Pharmaceutics. 2022 Sep 9;14(9):1911. doi: 10.3390/pharmaceutics14091911 (PMC9502491; doi:10.3390/pharmaceutics14091911)
Supplement: Supplementary file 1 [file pharmaceutics-14-01911-s001.zip › pharmaceutics-1826001-supplementary.pdf]

# Supplementary Materials: New data for Nebivolol after in silico PK study: focus on young patients and dosage regimen

**Table S1.** Demographic characteristics (mean ± standard deviation) of patients in each clinical study.

| Individual | Age (years) | BMI (kg/m$^2$) | Genotype | Ethnic | Study Number |
|---|---|---|---|---|---|
| 1 | 34.4 | 26 | EM | Caucasian | 1 |
| 2 | 25.7 | 24 | EM | Caucasian | 2 |
| 3 | 25.08 | 23.29 | EM | Caucasian | 3 |
| 4 | 29.67 | 24.17 | PM | Caucasian | 3 |
| 5 | 24.83 | 22.30 | EM | Caucasian | 4 |
| 6 | 24.83 | 22.30 | EM | Caucasian | 5 |
| 7 | 24 | 23.19 | IM | Asian | 6 |
| 8 | 24 | 20.10 | IM | Asian | 6 |
| 9 | 24 | 21.85 | IM | Asian | 6 |
| 10 | 20 | 20.26 | IM | Asian | 6 |
| 11 | 24 | 21.89 | IM | Asian | 6 |
| 12 | 25 | 22.87 | EM | Asian | 6 |
| 13 | 24 | 19.93 | IM | Asian | 6 |
| 14 | 24 | 20.71 | EM | Asian | 6 |
| 15 | 23 | 19.22 | EM | Asian | 6 |
| 16 | 25 | 21.08 | IM | Asian | 6 |
| 17 | 27 | 20.01 | EM | Asian | 6 |
| 18 | 23 | 19.73 | IM | Asian | 6 |
| 19 | 24 | 21.41 | EM | Asian | 6 |
| 20 | 24 | 21.16 | EM | Asian | 6 |
| 21 | 24 | 22.19 | EM | Asian | 6 |
| 22 | 22 | 19.90 | EM | Asian | 6 |
| 23 | 21 | 22.19 | IM | Asian | 6 |
| 24 | 23 | 22.53 | EM | Asian | 6 |
| 25 | 25 | 20.55 | EM | Asian | 6 |
| 26 | 24 | 23.63 | EM | Asian | 6 |
| 27 | 22 | 22.34 | EM | Asian | 6 |
| 28 | 24 | 21.62 | IM | Asian | 6 |
| 29 | 23 | 21.83 | IM | Asian | 6 |
| 30 | 25 | 21.00 | IM | Asian | 6 |

**Table S2.** Pharmacokinetics (PK) parameters (mean ± standard deviation) of patients in each clinical study.

| **Study Number 1** | |
|---|---|
| **PK parameters** | **n = 26** |
| $C_{max}$ (ng.mL$^{-1}$) | 8.02 ± 3.47 |
| *AUC* (ng.mL$^{-1}$) | 41.50 ± 29.76 |

| | |
|---|---|
| T*max* (h) | 1.32 ± 0.67 |
| K*el* (L/h) | ND |
| $t_{1/2}$ (h) | 11.03 ± 2.12 |
| **Study Number 2** | |
| **PK parameters** | **n = 23** |
| Cmax ($ng.mL^{-1}$) | 1.78 ± 1.17 |
| AUC ($ng.mL^{-1}$) | 12.09 ± 24.51 |
| Tmax (h) | 1.37 ± 0.88 |
| Kel (L/h) | 0.25 ± 0.30 |
| $t_{1/2}$ (h) | 6.65 ± 8.55 |
| **Study Number 3** | |
| **PK parameters** | **n = 43** |
| Cmax ($ng.mL^{-1}$) | 139.22 ± 20.80 (PM)<br>9.19 ± 8.91 (EM) |
| AUC ($ng.mL^{-1}$) | 5.24 ± 0.48 (PM)<br>1.66 ± 0.86 (EM) |
| Tmax (h) | ND |
| Kel (L/h) | ND |
| $t_{1/2}$ (h) | ND |
| **Study Number 4** | |
| **PK parameters** | **n = 18** |
| Cmax ($ng.mL^{-1}$) | 1.67 ± 0.69 |
| AUC ($ng.mL^{-1}$) | 10.38 ± 10.53 |
| Tmax (h) | 1.81 ± 1.19 |
| Kel (L/h) | 0.13 ± 0.09 |
| $t_{1/2}$ (h) | 7.05 ± 3.69 |
| **Study Number 5** | |
| **PK parameters** | **n = 18** |

| | |
|---|---|
| Cmax (ng.mL$^{-1}$) | 1.67 ± 0.69 |
| AUC (ng.mL$^{-1}$) | 10.40 ± 10.50 |
| Tmax (h) | 1.81 ± 1.19 |
| Kel (L/h) | 0.13 ± 0.09 |
| $t_{1/2}$ (h) | 7.05 ± 3.69 |
| **Study Number 6** | |
| **PK parameters** | **n = 24** |
| Cmax (ng.mL$^{-1}$) | 2.65 ± 1.33 |
| AUC (ng.mL$^{-1}$) | 18.21 ± 14.11 |
| Tmax (h) | 1.5 ± 0.9 |
| Kel (L/h) | ND |
| $t_{1/2}$ (h) | 12.52 ± 5.72 |

C*max* – maximum observed concentration; *AUC* – area under the curve from the time of dosing to the last measurable positive concentration; T*max* – time of maximum observed concentration; K*el* – elimination rate constant; $t_{1/2}$ – half-life time.

**Table S3.** Different basic population pharmacokinetic models of Nebivolol.

| Project name | Compartment | Error model | BIC |
|---|---|---|---|
| PK_N_01 | Two | Combined 1 | -278.87 |
| PK_N_02 | One | Combined 1 | 42.83 |
| PK_N_03 | One | Combined 1 | 128.53 |
| PK_N_04 | Two | Combined 1 | -170.82 |
| PK_N_05 | Two | Combined 1 | -275.68 |
| PK_N_06 | Two | Proportional | -401.07 |
| PK_N_07 | One | Combined 1 | 51.63 |

BIC: Bayesian information criteria; ΔOFV: Based on the BIC values, PK_N_06 (yellow) were the selected model for the study, due to their best description of the population pharmacokinetic profile.
